# Challenges and opportunities for digital twins in precision medicine from a complex systems perspective

**DOI:** 10.1038/s41746-024-01402-3

**Published:** 2025-01-17

**Authors:** Manlio De Domenico, Luca Allegri, Guido Caldarelli, Valeria d’Andrea, Barbara Di Camillo, Luis M. Rocha, Jordan Rozum, Riccardo Sbarbati, Francesco Zambelli

**Affiliations:** 1 Department of Physics and Astronomy “Galileo Galilei”, University of Padua, Padova, Italy; 2 Padua Center for Network Medicine, University of Padua, Padova, Italy; 3 Padua Neuroscience Center, University of Padua, Padova, Italy; 4 Istituto Nazionale di Fisica Nucleare, sez. di Padova, Italy; 5 DSMN and ECLT Ca’ Foscari University of Venice, Venezia, Italy; 6 Institute of Complex Systems (ISC) CNR unit Sapienza University, Rome, Italy; 7 London Institute for Mathematical Sciences, Royal Institution, London, UK; 8 Department of Information Engineering, University of Padua, Padova, Italy; 9 Department of Comparative Biomedicine and Food Science, University of Padua, Padova, Italy; 10 School of Systems Science and Industrial Eng., Binghamton University, Binghamton, NY USA; 11 Universidade Católica Portuguesa, Católica Biomedical Research Centre, Lisbon, Portugal

**Keywords:** Experimental models of disease, Preventive medicine

## Abstract

Digital twins (DTs) in precision medicine are increasingly viable, propelled by extensive data collection and advancements in artificial intelligence (AI), alongside traditional biomedical methodologies. We argue that including mechanistic simulations that produce behavior based on explicitly defined biological hypotheses and multiscale mechanisms is beneficial. It enables the exploration of diverse therapeutic strategies and supports dynamic clinical decision-making through insights from network science, quantitative biology, and digital medicine.

## Introduction

Precision medicine aims to deliver diagnostic, prognostic, and therapeutic strategies specifically tailored to individuals by explicitly accounting for their genetic information, lifestyle, and environment^1^, which are organized in a network structure^2^. The success of this approach relies on at least two fundamental and non-trivial assumptions: The first is that it is possible to predict the response of a patient to a specific treatment by means of computational, cellular, and organism-based models with reasonable accuracy. The second is that it is possible to use heterogeneous data sources (multiomics, electronic health records, individual and social behavior, and so forth) to build massive databases with enough statistics to stratify a population and characterize the distinctive features of clinical interest^3^.

It is not surprising that the field of precision medicine is growing^4,5^, attracting the interest of both national health systems for investments^6^, and scholars, who span a wide range of disciplines, from molecular biology to computer science, medicine, physics, and engineering. Nevertheless, precision medicine, with its revolutionary promises, is usually associated with clinical genomics^7^ and multiomics^8^, with a strong focus on the idea that combining heterogeneous, multi-scale sources of data will lead to timely predictions about individual medical outcomes. Recently, attention has shifted to the possibility of integrating such molecular data with traditional^9–11^ and non-traditional^12^ data sources of clinical relevance into a multiscale predictive modeling methodology. This leads to the creation of a *digital twin*, and it allows for testing therapeutic strategies in-silico with the ultimate goal of maximizing successful treatments and outcomes in vivo.

**Defining digital twins in precision medicine** The first pioneering precursors of digital twins for personalized medicine came out in the early 2000s and proposed the idea that models of the human body for specific patients could improve clinical practices. They also pointed out challenges that are still valid, such as the need for a structure to handle multiple-source data integration^13^ and the importance of having solid mathematical models that can describe the system at the desired level of precision^14^. In recent years, medical digital twins have experienced a huge increase in interest, with the birth of many programs devoted to them^15,16^. Two of the most significant successes in the field are the “artificial pancreas” of the ARCHIMEDES program on diabetes^17^ and the mechanistic models of the heart used for cardiovascular disease monitoring and prevention^18,19^. Recent research emphasizes the potential of having a comprehensive model of the human body that could reflect the possible consequences of a perturbation, such as a viral infection^20^ or taking a drug^21,22^, on a specific patient. To actually implement these models, now a decade from the first proposals^23^, network and complex systems are starting to be considered^15^. Meanwhile, approaches based on artificial intelligence (AI) and machine learning have been widely adopted despite some limitations and critical aspects^24^.

Given the currently broad spectrum of definitions and applications, it is important to set the operational definition that we adopt throughout this paper Box 1, Box 2.

Broadly speaking, a digital twin exchanges data with its real-world counterpart, synchronizing inputs and outputs; they operate synergistically, with the digital twin informing, controlling, aiding, and augmenting the original system.

Indeed, a digital twin is a virtual replica of a physical system, object, or process. It is designed to reproduce the behavior, conditions, and responses of its real-world counterpart in real time or near-real time. However, while a digital twin excels at reproducing these behaviors, it does not inherently explain them. The situation is similar to that of the maps, as noted by Borges^25^; if we want to build the best possible twin of a system we have to replicate the system itself, which does not necessarily advance our understanding. Explanation requires understanding why processes and phenomena happen. Although a digital twin can show what is happening, it does not provide underlying reasons or insights unless specific analysis tools or models are integrated. For example, it may reproduce a failure in the system under analysis, but it does not automatically explain the root cause unless additional diagnostics or analytics are applied. Unfortunately, causes can be intertwined in a network of other networks. Accordingly, these objects are best described and understood as complex systems. Indeed, explanation often involves understanding cause-and-effect relationships, system interdependencies, or emergent properties that may not be obvious from reproducing real-time behavior.

Interest in digital twins has also exploded well beyond medicine due to increasing access to memory, computational power, and massive data gathering. Digital twins have also been applied to cities^26^, primarily to simulate the intricate infrastructural configurations and products^27^, by leveraging contemporary technologies such as data analytics, IoT-driven physical modeling^28^, machine learning, and AI. For cities^29^, it may be far more efficient to consider the emerging behavior arising from the intricate web of relationships, processes, and correlations that characterize the complex adaptive system^30–32^ than to produce a mere copy of it.

**Open challenges** We consider current methodological advances and challenges in these other fields to highlight the existing challenges in precision medicine. Despite promising opportunities to create a digital copy of every individual, there are some caveats to address to allow for personalized analysis and testing of individual-specific therapeutic strategies^15,21,22^.

On the one hand, if digital twins must be designed to be perfect replicas of individuals, then the amount of data required vastly exceeds our present, and even future, possibilities. The gigantic number of intervening functional units, from biomolecules to cells, makes any analytical or computational approach impossible. Even in the ideal case that a perfectly functioning computational framework was technologically accessible, the nonlinear dynamics of interacting biological units lead to emergent phenomena that cannot be simply simulated or predicted, which is a hallmark feature of complex systems^33,34^. Because of this, recent advances in predictive biology are based on building models of increasing complexity to reproduce only the most salient characteristics of complex biological processes in engineered and natural populations^35^.

On the other hand, human patients have their own dynamical response to internal dysfunctions or differentiated coupling to the environment. This includes individual histories of host-microbiome and host-pathogen interactions, which might jeopardize any predictive model. Even more widely, the full individual exposome includes all past exposure to specific multi-scale environmental factors, such as diet and reactions to stressful biochemical or social conditions^36,37^. While the causal mechanisms in multiomic regulation can be partially reconstructed and accounted for, the full individual exposome is almost impossible to replicate or reproduce with a digital twin.

**Fig. 1:**
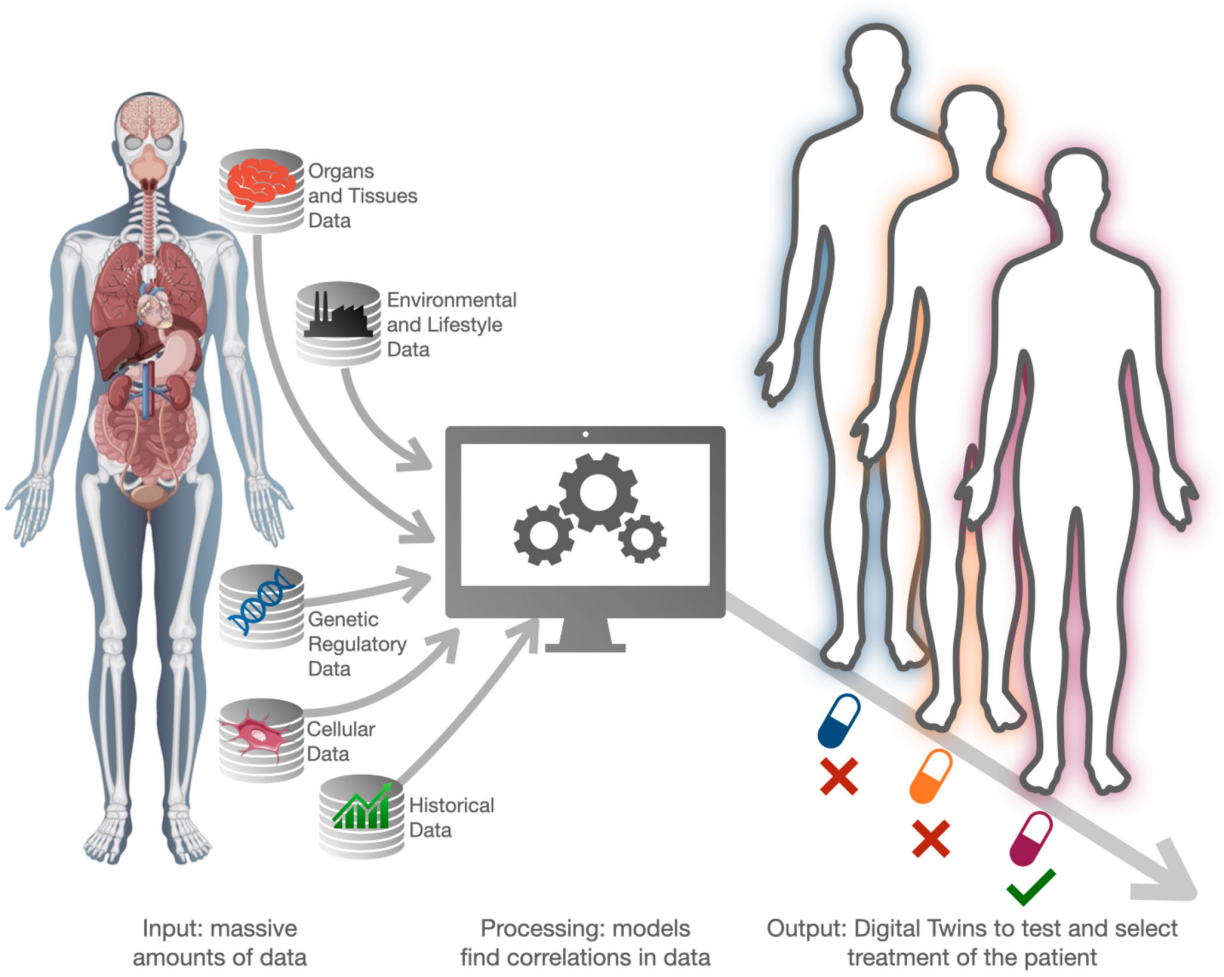
Precision medicine standard approach for digital twins. The framework relies on using large-scale heterogeneous data sources (pre-clinical, clinical, environmental, lifestyle, etc.). This massive database can be used in sophisticated computational models (such as deep learning), while relying solely on statistical data analysis to construct a series of digitalized instances–the digital twins–of a patient, which can then be used to test one or more therapeutic strategies for clinical decision-making. Human body design by Freepck and osteocytes from Servier Medical Art (smart.servier.com).

We have made great strides in capturing the exposome via the collection of new types of data from sources such as mobile devices^38^ and social media^12^. However, even in the most ideal cases, unknown factors such as the level of disease progression and unmeasured lifestyle changes can lead to a broad set of distinct outcomes that make the design of digital twins very sensitive to the quantity and accuracy of input data. This technology may struggle to adapt and accurately predict these dynamic changes, which would lead to sub-optimal personalized treatment recommendations. These potential issues can dramatically hinder the purpose of digital twins, which might suggest that only methods based on advanced statistical data analysis, such as machine learning, are viable. This is not the case, however, because such methods provide predictive models that (i) do not easily generalize to situations and conditions for which they have not been trained, and (ii) might recommend clinically sub-optimal solutions when they retrieve multiple outcomes that they have ranked similarly (Fig. 1).

Therefore, a more comprehensive approach based on methods that capture the essential features of complex interconnected and interdependent systems^39,40^ at many scales is needed. This approach must (i) reduce the dimensionality of the problem of interest by identifying the key biological, clinical, and environmental variables needed for an adequate description on short time scales; (ii) characterize the conditions under which a complex adaptive system like the human body (or even a cell line) can be simulated by a digital twin in terms of separated components or sub-systems; and (iii) provide a transparent computational framework for testing actionable intervention strategies that are based on what-if scenarios and clinically relevant, model-informed, data-driven, and evidence-based questions.

In short, this calls for a more holistic and quantitative approach based on the complex adaptive nature of every patient rather than a mere replica of their salient aspects for statistical analysis.

Box 1 Definition of digital twins in precision medicine A digital twin is an in-silico framework that replicates a biological cell, sub-system, organ, or a whole organism, with a transparent predictive model of their relevant causal mechanisms and response to interventions.

Box 2 Operational definitions of context-specific terms **Precision medicine:** Also referred to as personalized medicine, this refers to a medical approach that tailors healthcare to the individual characteristics of each patient, based on their genetic makeup, environment, lifestyle, and other personal data.

**Network model:** A mathematical model to represent a complex system as a set of entities (nodes) connected pairwise by links (edges) to indicate association or interaction. Nodes and edges can be of very different natures, such as, respectively, proteins and physical interaction between them for protein-protein interaction networks, neurons and synapses for neural networks, species and predation interactions for ecological networks.

**Organoid:** A self-organized 3D tissue that is typically derived from stem cells (pluripotent, fetal, or adult) and mimics the key functional, structural and biological complexity of an organ^146^.

**Exposome:** External factors (environmental, social, etc.) that an individual is subjected to throughout life that may be related to their state of health.

**Multiscale models:** Mathematical models that describe interactions within and between components of a system with spatial or temporal scales that differ by orders of magnitude. For example, in gene expression, the folding and unfolding processes of the chromatin typically occurs over hours to days, while transcription occurs over minutes.

**Agent-based model:** A computational simulation that represents a system as a collection of autonomous proxies (e.g., representing cells or individuals) interacting within a defined environment, each with its own characteristics, rules, and behaviors that collectively give rise to complex system dynamics.

**Boolean automata:** An agent in a discrete dynamical model that can take one of two states-ON or OFF. It determines its next state based on the values of pre-specified input variables, which are combined using a fixed Boolean logic function.

**Boolean networks:** A collection of interconnected Boolean automata in which each automata’s (node’s) inputs correspond to the states of other automata (parent nodes or regulators).

## Multiscale modeling in health and disease: from genes to systems

To design effective digital twins, accounting for the multiscale nature of biological systems is of paramount importance. Recent progress in the study of complex systems, especially those with interconnected and interdependent structure, dynamics, and function, provide promising ground for figuring out and illustrating how diverse functional units and sub-systems interact at different scales. Indeed, in addition to extracting multiscale molecular details from large omics datasets (e.g., transcriptomic, genomic, metabolomic, and microbiomic), we can now extract large-scale human behavior data of biomedical relevance from social media, mobile devices, and electronic health records, including new patient-stratification principles and unknown disease correlations^9,11,12,38,41–43^. Accordingly, the holistic integration and analysis of such multiscale data sources constitutes a novel opportunity to further improve personalization by including the exposome in the study of multilevel human complexity in disease^42,44^. This can be used to inform more accurate models for predictive purposes in biomedicine^35,43,45–47^.

### Processes at the intracellular scale

At the smallest scale, gene regulatory networks are systems of interacting genes and their regulatory elements within a cell that control the level of gene expression. In these networks, the nodes usually represent genes and the edges usually represent regulatory interactions between them. They describe the timing, spatial distribution, and intensity of gene expression, thereby orchestrating various cellular processes such as development, differentiation, and response to environmental stimuli^48–51^. A protein-protein interaction (PPI) network captures distinct types of interactions (e.g., physical contacts) between proteins in a cell. In PPI networks, nodes represent individual proteins, and edges encode interactions between them, which can be transient, as in signal transduction, or more stable, as in protein-complex formation^52,53^. PPI networks provide insights into cellular processes, functional associations, and the modular organization of proteins, and analyzing the structure and dynamics of PPI networks helps uncover the underlying principles of cellular organization and function^46,54–60^. Metabolic networks^43,61,62^ map out the biochemical reactions that occur within an organism, detailing how individual metabolites are synthesized, degraded, and interconverted^63^. These networks are either composed of nodes, representing metabolites, and edges, indicating the enzymatic reactions facilitating the transformation from one metabolite to another, or bipartite networks, where nodes are chemical species on one side and reactions on the other. In the latter representation, the web of metabolic interactions is intricately woven, while in the former, it is more straightforward. Beyond individual reactions, these networks highlight the interconnected nature of metabolic pathways, which reveal redundancies, feedback loops, and regulatory mechanisms that maintain cellular homeostasis.

Intracellular networks have time-evolving states that describe which genes are active, which proteins are present (or phosphorylated, oxidized, ubiquitinated, etc.), the concentrations of metabolites, and so on. State evolution is often studied using ordinary differential equation (ODE) models, which can be fit to match experimental state and kinetic data^64^. In many cases, the available data is insufficient to fully constrain the parameters of an ODE model. Also, it is often the case that the underlying biological dynamics is of a threshold nature^65^. In these cases, a discrete causal model, such as a Boolean network (or multistate automata network, more generally), may be appropriate^66,67^. In a Boolean network, the state of each node in the intracellular network is binarized: a gene is either active or inactive, and the active form of a protein is either above some unspecified threshold of abundance or below it. The binarized states change in time according to logical (Boolean) update functions; that is, each network node is an automaton^68^. The causal effect of various interventions (e.g., drugs) can be evaluated by manipulating the states of individual nodes and observing the resulting dynamics.

Because Boolean automata can be grouped to model variables with more than two states, the approach is widely applicable for modeling cellular components with various levels of activation, such as the proportion of cells that enter apoptosis in breast cancer cell lines^69^. Indeed, a common application of these models is in studying the effects of combinatorial drug interventions, particularly in the context of cancer^69,70^. To serve as a component of a digital twin, Boolean networks must reconcile their discrete time steps with physical time. This is often done by updating node states asynchronously according to tunable node transition rates, essentially treating the dynamics as a continuous Markov process^71^. This approach has been applied, for example, to suggest personalized drug therapies for prostate cancer patients using personalized Boolean network models^72^. One important advantage of Boolean or multistate automata networks is that they allow the precise characterization of polyadic relationships^65,68^. In other words, they are a type of higher-order network that can capture multivariate associations and interactions beyond the pairwise relations afforded by graphs in typical network science praxis^73,74^. In addition, this discrete dynamics approach has been used to infer important dynamical pathways in multilayer networks, which is another case of a higher-order network^40^. For instance, tying molecular factors (from multiomics, brain, and retinal imaging data) to clinical phenotype (from patient data) in multiple sclerosis^47^. This is an exciting avenue that allows complex regulatory dynamics to be studied on static multilayer networks obtained from heterogeneous data sources. Thereby, each node can integrate incoming signals differently, which goes well beyond the typical analysis via spreading or information dynamics on networks.

It is important to note that automata network models can typically be greatly simplified by reducing dynamically redundant interactions^68^, which are due to the ubiquity of canalized dynamics in biology^75,76^. This results in scalable causal models capable of uncovering actionable interventions, conditioned on different input assumptions, in a transparent manner^65,68,74^. Boolean networks are especially amenable to causal analysis because they can be converted to simplified causal representations (according to Boolean minimization criteria)^65,68,77,78^. They stand in stark contrast to the black-box predictions of traditional machine learning methods and tallying the outputs of Monte Carlo simulations of large dynamical models (including non-simplified Boolean Networks).

Thus, automata network models—whose parameters can be inferred and validated from perturbation experiment, multiomics, and exposome data—are ideal components to consider for the top level of digital twins. They synthesize the large-scale underlying data into simplified, explainable, causal networks amenable to investigating actionable interventions. Indeed, these features show how this modeling approach directly responds to the needs of the digital twin approach identified in the introduction: dimensionality reduction, scalable modularity, and transparency.

### Processes at the whole-cell scale

Whether discrete or continuous, the dynamics of intracellular networks can be coupled with each other and with physical processes to produce whole-cell models. These models attempt to describe the whole genome, proteome, and metabolome of a cell over the course of its life cycle in a fine-grained dynamical model^79^, as was first demonstrated in the human pathogen *Mycoplasma genitalium*^80^. More recent efforts have been focused on identifying minimal genomes^81^ or modeling organisms with larger genomes, such as *E. Coli*^82^. Currently, the biomedical application of such detailed models is limited by the enormous effort required to construct them. Fortunately, to build a medically relevant digital twin, it is often the case that only specific processes need to be incorporated. Narrowing the focus of the model at the cellular level makes model construction and personalization more feasible, lowers computational barriers, and facilitates embedding these models into multicellular models, as in^83^.

An interesting focus arises from single cell data analysis. Even cells of the same type exhibit different system state and expression profiles in tissues, which are complex multi-agent systems made-up of multiple subpopulations of cells. They are spatially and temporally organized, able to communicate and interact with each other and orchestrate self-assembly and response to stimuli as a whole. This is fundamental in many biological contexts, such as early embryonic development and tumor etiology, where different cells are characterized by distinctive genetic mutations or expression profiles. These differences are regulated by cell-to-cell communication and underlie complex dynamic responses characterizing healthy and pathological tissue development^84^.

An example of how the interaction between cells can be modeled to describe emergent behaviors is the study of the interaction dynamics between immune and tumor cells in human cancer using agent-based models. By coupling a discrete agent-based model with a continuous partial-differential-equation-based model, these models capture essential components of the tumor microenvironment and are able to reproduce its main characteristics. Each tumor is characterized by a specific and unique tumor microenvironment, which emphasizes the need for specialized and personalized studies of each cancer scenario. Recently, a model of colon cancer has been proposed that can be informed with patient transcriptomic data^85^. It would be interesting to extend this model by informing it through methods that infer cellular communication^86–89^, which has the advantage of characterizing the tumor environment more specifically by defining the probability of an agent’s action in response to received communication.

### Processes at the intercellular scale and beyond

At the tissue scale, systems that must be considered include neural, cardiovascular, and respiratory, but also their dysfunctions, such as cancer. Whether investigating the intricate networks within the human brain or the simpler wiring maps of organisms like *C. elegans* or *Drosophila Melanogaster*, the objective remains consistent: to elucidate the interconnections and organization of neurons and regions at the mesoscale. Several studies have focused on examining neural connections within an organism’s brain, commonly referred to as the connectome^90^. Functional imaging techniques are utilized to explore the relationship among activities in specific brain areas^91^. The analytical and computational tools from network theory allow us to build maps of structural and functional connections, revealing characteristics of complex networks-such as small-world topology, highly connected hubs, and modularity-that manifest at both the whole-brain and cellular scales^92–94^. The human brain is an emblematic case study for the design of ambitious computational models toward developing digital twins. Nevertheless, despite the aforementioned significant advancements, the explicit goal of building a realistic computer simulation of the brain within a few years has not met expectations^95^.

**Fig. 2:**
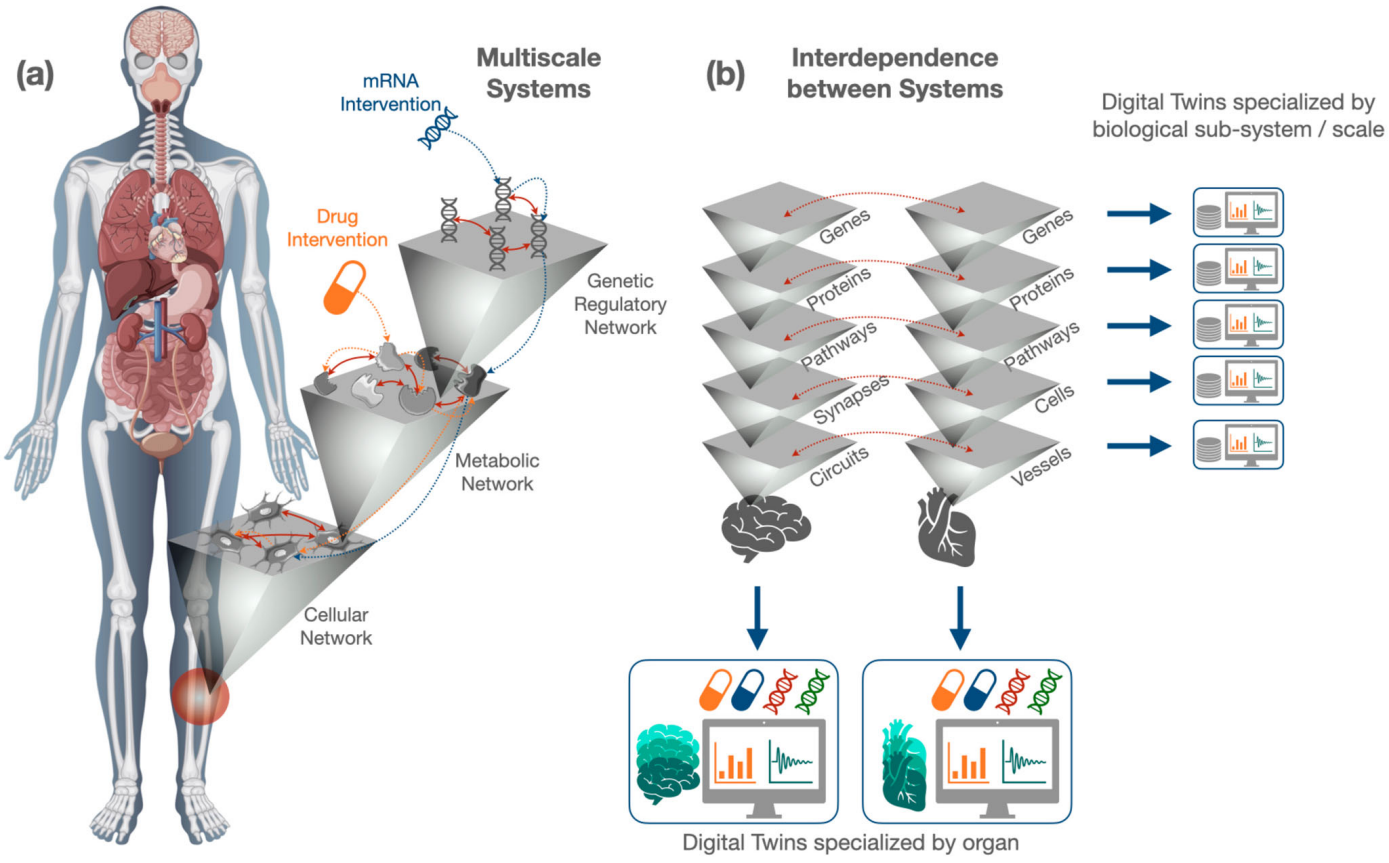
Multiscale and network modeling for digital twins. **a** Once the potential source of a dysfunction is identified, multiple biological systems might be involved across different spatial and temporal scales. Treatments based on, e.g., mRNA therapy or classical drugs, usually target biological units at a specific scale. However, treatment effects might propagate to other units at the same scale or across scales due to the presence of interactions and interdependencies among biomolecules and functional sub-systems, such as cells. **b** A multiscale illustration of the interdependent sub-systems related to the function of distinct organs. Each scale can be simulated by a specialized digital twin, or multiscale integration can be simulated by a more complex, but still specialized, digital twin. The effects of distinct treatments can be analyzed on several distinct instances of the digital twin using a model-informed and data-driven search. Human body design by Freepik and osteocytes from Servier Medical Art (smart.servier.com).

Overall, sub-systems are part of a broader complex, adaptive, interdependent system of systems which are organized in hierarchies of increasing complexity with modular organization^96^. This is a fact well recognized for at least half a century and summarized in the Jacob’s statement that “every object that biology studies is a system of systems”^97^. Sub-systems exchange information (e.g., in terms of electrical, chemical, and electrochemical signals) to regulate each other and operate out of equilibrium^98–100^. Consequently, considering sub-systems in isolation from one another provides an incomplete representation of each sub-system and leads to inaccurate models and predictions of biological processes. A partial solution to this problem comes from the statistical physics of multilayer systems, which allows each scale to be described by a *level* of organization, and each level to be characterized by multiple context *layers*^40,101^. Levels can be interdependent^39,102^ while also being characterized by different contexts. In the case of biological systems^103^, this is reflected in the distinct types of interactions among the same set of biomolecules or the distinct channels available for cell-cell communication, and also in the interdependence between distinct systems such as the cardiovascular and nervous systems (Fig. 2). This web of interconnections and interdependencies involving diverse and heterogeneous functional biological units across scales plays a pivotal role in human health, and it is plausible to associate their dysfunction with disease states^104,105^.

While gathering, ingesting, analyzing, and accessing in real-time all the necessary multiscale data for multilayer network models will remain a challenge for the foreseeable future, data science already provides scalable methods for federating and visualizing such heterogeneous data^106^. One exciting approach is the construction of knowledge graphs to represent biomedical concepts and relationships extracted from human-annotated databases or automated pipelines^107^. These have been used, for instance, to design epilepsy patient self-management tools that link disease factors from molecular and pharmacological databases, clinical practice and epidemiology from electronic health records, and even exposome factors extracted from social media^108^.

Successful use cases of multilayer networks also include applications from personalized multiomics, where information from mRNA, miRNA, and DNA methylation has been integrated into a multilayer structure to unravel the peculiar microscopic and mesoscopic organization in Chronic Obstructive Pulmonary Disease^109,110^, rare diseases such as Congenital Myasthenic Syndromes^43^ and Medulloblastoma^111^. Multilayer networks provided fertile ground for characterizing diseases while accounting for multidimensional factors such as molecular interactions and symptoms^112^, for building integrated models of signaling and regulatory networks^113^, and for analyzing functional organization in the human brain for subjects affected by psychiatric disorders such as Schizophrenia^114^ and neurodegenerative diseases such as Alzheimer’s^115–117^.

## Challenges in multiscale modeling

### Limitations in data-driven approaches to digital twins in precision medicine

Multiscale modeling of biological systems presents formidable challenges, primarily due to the intricate and redundant networks of interactions and interdependent processes taking place. They unfold across different scales, from molecular (microscopic) to organismic (macroscopic) levels. These systems are characterized by dynamic processes that operate far from equilibrium; they exchange various types of signals—for example, chemical, electrochemical, and more – thereby creating a complex ecosystem of interlinked dynamical processes^35^. Such complexity poses significant difficulties in developing models that are both consequential and coherent, while avoiding extremes like reductionism, which assumes that sufficient computational power can simulate an entire organism, or oversimplification, which relies excessively on abundant data to sidestep the need for intricacy.

Moreover, biological systems are inherently adaptive, adjusting dynamically to environmental changes^118^. This adaptiveness is crucial for accurately simulating the impact of external factors such as therapeutic interventions or changes in environmental conditions, like pollution^119^ or alterations in food sources^120,121^. Responses to these changes start at the cellular level, influencing gene expression, post-translational modifications to proteins, metabolic fluxes, and so on, leading to sometimes irreversible epigenetic changes^43,122,123^. These responses ultimately extend to organs, higher biological systems, and overall phenotype response through complex signaling pathways. Such adaptive complexity, represented in now available genomic, epigenomic, and other multiomic data^123^, cannot be accounted for by statistical methods only. It must be integrated into causal models to accurately reflect the biological response to external stimuli within the spatial and temporal scale of interest and to generate causal hypotheses^124^.

In the broader context of precision medicine, integrating digital twins that reflect these multiscale, multiomics and adaptive features poses even more challenges. The models often employed are predominantly phenomenological, focusing more on observed phenomena rather than the underlying mechanisms. This approach results in a significant gap in mechanistic understanding, which is essential for bridging various biological scales effectively. Cities face similar multiscale integration challenges^29^ and require a similar framework to address the complex interplay of different components within the living system, which potentially guides the development of more effective biomedical models. Accordingly, a crucial preliminary step is establishing reliable and widely accepted standards for defining and measuring relevant quantities, along with their associated errors. The medical field often struggles with standardization due to the significant impact of individual physician expertise on diagnosis and treatment, which explains the challenges in creating uniform procedures. Digital twins can help overcome these obstacles by providing a shared framework of quantifiable parameters and standardized measurement practices. However, even with carefully defined protocols, some key properties may remain elusive. In such cases, network reconstruction techniques can assist by inferring the structure of biological networks from incomplete or noisy data. Because medical networks are rarely fully observable, reconstructing missing links or predicting unobserved connections becomes essential for a comprehensive system description^125–129^.

### Toward hypothesis-driven generative models

By leveraging properties of network topology, such as degree distributions and community structures within statistical ensemble frameworks, we can enhance the accuracy of our network predictions, ultimately improving our mechanistic understanding across biological scales. Note, however, that standalone data-driven models heavily rely on the completeness and quality of data, while missing, conflicting, or poor-quality data can lead to inaccurate predictions. In the context of a digital twin, hypothesis-driven models could leverage prior knowledge or established theories to fill gaps, even in the absence of complete data, thus providing a clear advantage. Genetic-algorithm inference of qualitative models provides a computationally efficient way to explore multiple cellular contexts and to ultimately reconcile apparently contradictory experimental measurements without being overly sensitive to small changes in experimental design^130–132^.

We use the term “hypothesis-driven generative models” to refer to mechanistic simulations that produce behavior based on explicitly defined biological hypotheses and mechanisms. These models simulate biological processes by incorporating known or hypothesized interactions and pathways, which enables the generation of predictive and explanatory behaviors grounded in mechanistic understanding of the intervening physical, chemical, and biological processes. This approach differs from data-driven generative AI models, such as those based on variational autoencoders or generative adversarial networks, which learn patterns from data without necessarily incorporating underlying biological mechanisms.

By critically analyzing these challenges through the lens of complexity science, we can better understand and possibly overcome the hurdles in creating cohesive and predictive multiscale models that are crucial for the future of biomedical research and therapeutic development. In the case of interconnected systems at a given scale, we can introduce a suitable object, named multilayer adjacency tensor ${M}_{j\beta }^{i\alpha }(t)$, to operationally encode all the interactions at time *t* between a biological unit *i* (e.g., a single protein or a protein complex) in a layer *α* (e.g., a class of biological processes or a pathway) and another biological unit *j* (e.g., another protein or a metabolite). The framework is so general that it can also include potential cross-layer structural interactions. In fact, due to the high number of interacting units (such as biomolecules, cells, etc.), biological modeling often assumes such deterministic processes, for example, that reactions occur at constant rates, compartmental interactions are fully-mixed, or mean-field approximations apply. Therefore, even at some good level of approximation, the dynamics of some quantity **x**(*t*) of interest, for example, the concentration of metabolites or the population of some species such as cancer cells or bacteria, might be described by multilayer differential equations^40,133^ like

1 $$\frac{\partial {x}_{j\beta }(t)}{\partial t}={f}_{j\beta }({x}_{j\beta },t)+\sum _{i}\sum _{\alpha }{g}_{j\beta }\left[{M}_{j\beta }^{i\alpha }(t),{x}_{i\alpha }(t),{x}_{j\beta }(t),t\right],$$

where *f*_*jβ*_(⋅) is a function only of the variable *x*_*jβ*_(*t*) corresponding to a specific unit *j* in a specific context or layer *β*, and *g*_*jβ*_(⋅) is a function that accounts for the interactions between pairs of units, that is, for the effects due to the intervening networks.

It is remarkable how such a simplified deterministic framework can model different phenomena of medical interest. Examples include responses to clinical treatment, cell activity stimulation, or even protein production, which are triggered by specific factors, such as basic chemical reactions, pH levels, drug concentration, or specific mRNA targets^134,135^. In light of these simple arguments, it might be tempting to rely only on such deterministic approaches–based on sets of differential equations, such as Eq. (1), or on agent-based modeling – to predict the behavior of a therapeutic intervention. After all, if we have systematic cause-effect relations linking interventions to biological and clinical outcomes, it would be enough to calibrate our models on the specific features of a patient to determine their response to treatments and potentially cure a disease.

However, in complex and variable environments such as a living organism, adaptiveness, randomness, and biological noise might affect the model outcomes. Still, adaptiveness can be reflected by such simplified models. If we indicate with *u*_*jβ*_(*t*) some external input signal or control applied to a biological system, and with *Θ* the set of parameters that dynamically change based on the system’s states or external inputs, then a more general model at a given scale could be formalized as

2 $$\begin{array}{ll}\frac{\partial {x}_{j\beta }(t)}{\partial t}\,=\,{f}_{j\beta }({x}_{j\beta },t)+\sum\limits_{i}\sum\limits_{\alpha }{g}_{j\beta }\left[{M}_{j\beta }^{i\alpha }(t),{x}_{i\alpha }(t),{x}_{j\beta }(t),\Theta ,{u}_{j\beta }(t),t\right]\\\qquad\qquad \frac{\partial {M}_{j\beta }^{i\alpha }(t)}{\partial t}\,=\,\ell ({M}_{j\beta }^{i\alpha }(t),{x}_{i\alpha }(t),{x}_{j\beta }(t),\Theta ,{u}_{j\beta }(t),t)\\\qquad\qquad \frac{\partial \Theta (t)}{\partial t}\,=\,h({x}_{i\alpha }(t),{x}_{j\beta }(t),\Theta ,{u}_{j\beta }(t),t)\,\text{,}\,\end{array}$$

which is much more complicated than Eq. (1), but it can be managed from a computational point of view. The integration of randomness and biological noise, which remains a challenge in mechanistic models, is further discussed in the following section.

### Limitations in multiscale deterministic modeling

Noise may be inherent to one or more aspects of the involved systems, for example, in the form of biochemical and electrochemical variability. Noise may also be linked to specific mechanisms altered by internal or external perturbations, such as virus-host interactions, environmental changes, and so on. Accordingly, when to include the effects of noise depends on the scale and impact of the biological process being modeled. For instance, including DNA replication errors for the analysis of short-term effects of a therapeutic drug might not add relevant biological or clinical insights, but would add undesirable complexity to the model. Another emblematic case is the use of discretized structures, such as networks, to model processes that are manifestly continuous (e.g., in space). Under such conditions, using complex networks introduces a level of sophistication that is not necessary to gain insights about a biological process.

Noise sources introduce an additional level of stochasticity that cannot be easily taken into account by statistical models, even the most sophisticated ones based on machine learning. Nevertheless, what is usually assumed to be a bug might be a feature: for other complex systems in nature, stochasticity is indeed structured and can lead to self-organized behaviors and processes^136–138^. The theory of nonlinear dynamical systems and the statistical physics of complex networks provide suitable theoretical and computational frameworks to model such complex biological phenomena^100^. They should be considered essential ingredients in designing reliable digital twins, either specialized or not, for any living organism.

The most important obstacle to describing realistic biological systems is how to incorporate multiple dynamic processes across the multiple intervening scales. The difficulty is primarily due to the diverse nature of the laws governing these processes at each scale. One significant technical challenge is effectively bridging these scales. This involves not just scaling up or down the processes, but also ensuring that interactions between scales are accurately captured. This might involve developing intermediate models or using scale-bridging techniques like homogenization or coarse-graining, which themselves can introduce approximation errors or require simplifications that might affect model accuracy. While some models are based on fundamental laws—such as reaction-diffusion processes for chemical networks—other models are genuinely phenomenological. Reconciling the dynamics of such different natures is challenging, since the latter class of models might not be suitable to capture novel phenomenology. This problem can be solved only partially by developing more fundamental models because biological processes are characterized by emergent phenomena that cannot be directly deduced even with full knowledge of their units and interactions^30–33^. To overcome this problem, we must simultaneously account for the evolution of the system according to dynamics similar to those in Eq. (2) and the fact that the underlying mechanisms can change while satisfying the constraints imposed by physics and chemistry, which requires meta-dynamical models^139^.

Additionally, multiscale models often require extensive parameterization, which can be difficult when experimental data are scarce at certain scales. Validating these models across all scales can be exceptionally challenging. This happens especially when direct observations or experiments at certain scales are not feasible or when they provide, at best, indirect measurements (such as correlations) about the phenomenon of interest that requires an adequate inferential framework^127^.

Furthermore, models should be able to propagate perturbations from one scale to another to realistically mimic the behavior of a living organism. As previously discussed, the possibility that a perturbation at the lowest scale (e.g., a random mutation or an mRNA intervention) can alter biological processes at larger scales is a mandatory feature for any reliable design of a digital twin.

## Discussion and outlook

Innovative approaches for model integration within digital twins have huge transformative potential in precision medicine by enabling a synergy between generative modeling, advanced AI and machine learning techniques, and traditional biomedical insights. The fusion of these techniques, rather than the choice of a specific one, is expected to facilitate the development of new frameworks for multiscale modeling. This is pivotal in capturing the intricate dynamics of pathogenesis in humans. Through these frameworks, the overarching goal is to resolve the challenges we have identified and significantly enhance the accuracy and clinical relevance of digital twins beyond inductive modeling via advanced statistics.

### From black to transparent boxes

On the one hand, the integration of mechanistic models into digital twins also addresses the challenges of parameter indeterminacy and overfitting, which are prevalent in systems characterized by vast parameter spaces. Digital twins constrain these spaces by, for example, the coarse-grained dynamics afforded by multiscale automata network models that synthesize large-scale data about biological mechanisms. In so doing, digital twins not only gain in robustness and explainability but also offer a more reliable foundation for the simulation of therapeutic outcomes, thereby increasing their utility in clinical practice. This activity can bring about a mechanistic clinical decision support system, that is, a type of decision support tool in healthcare that uses mechanistic models to assist clinicians in making medical decisions. Mechanistic models are based on an understanding of the underlying biological, physiological, or physical mechanisms that govern the behavior of systems in the human body. These models use known principles, from biochemistry, physics, population dynamics, and systems theory to predict outcomes or provide interpretations for clinical data. Mechanistic models are built on a theoretical understanding of how biological systems function. They often use mathematical equations to represent physiological processes such as blood flow, metabolism, or drug dynamics. Of course, these methods need to be tailored to each patient, and for this purpose, the methods of machine learning and analysis of each patient’s past behavior are of the utmost importance^140^.

On the other hand, it is also worth discussing what is missing in current technologies and techniques developed for the same aim. For instance, a critical advantage of digital twins over state-of-the-art non-computational models, such as organoids^141^, is their ability to simulate complex, interdependent processes across multiple biological scales effectively. They also provide explanatory and causal understanding and control at relatively small costs. Indeed, we should consider the full spectrum of digital twins. They are not restricted to whole organisms, but can also be used to model cell lines, sub-systems and organs. This makes them an exciting alternative or complement to organoids. It is certainly feasible to compare, for instance, digital twins of cancer cell lines^142,143^ with organoids synthesized for the same cell lines^144^. As discussed next, in this type of scenario, digital twins provide various advantages that need to be considered.

### The advantages of mechanistic digital twins

Organoids can be engineered, using the power of modern synthetic biology^145^, to recapitulate features of the function and response of complex biological mechanisms of the corresponding in vivo target, but they have important limitations. For one, reproducibility is a major bottleneck^146^. However, digital twins can excel at this, especially if built under an open-source framework. Additionally, organoids do not yet capture the entire physiological repertoire of cell types, or even the behavior that is relevant for a particular disease. This means, for instance, that the response to drugs or other interventions needs to be studied for organoids per se, separately from the in vivo target. They also have a relatively limited range of heterogeneity in responses, but a broader range is needed to develop truly personalized digital twins^146^. Finally, while organoids are more direct analogs of biomolecular mechanisms, they cannot incorporate simultaneously the multiple scales and historical information about patients, including the microbiome and exposome, which are major factors in complex conditions such as cancer, depression, and many chronic diseases.

This is where the comprehensive multiscale network- and data-driven digital twin approach is particularly crucial. Many complex diseases unfold across various multiomic sub-systems and exposome histories. Modular computational architectures that can synthesize and integrate multiple subsystems as separate network layers or agent-based models are well within the realm of possibility. They might require a robust non-specialized digital twin, effectively integrating different specialized ones, to accommodate complex interactions and interdependency of biological and exposome processes. While non-specialized digital twins may not allow individual patient precision, the approach could still increase precision for specific cohorts within the whole population. For diseases with more circumscribed features, however, specialized digital twins might offer precise intervention strategies and outcome predictions that could perform as well or better than those based on organoids.

Another remarkable advantage of digital twins is that they allow a scenario-based modeling approach for actionable interventions—akin to strategies routinely used in epidemic modeling for policy decision-making^147^—that enhances their applicability and safety in clinical settings. This method avoids the standard pitfalls of an oracle-like predictive model by allowing for exploration via direct simulation of multiple clinical scenarios, thereby providing a robust tool for decision support in personalized medicine. It requires the crucial integration of massive data sets about disease or treatment progressions. This provides reliable statistical samples that can be stratified to approximate the characterizing features of a patient, and to validate the output of models. Therefore, the expected model-informed and data-driven output would not be a unique therapeutic strategy or an intervention, but a whole spectrum of alternatives with the advantages and disadvantages of each plausible strategy outlined to inform human decision-making.

### Explainability and falsifiability via complex systems science

Digital twins have the potential to revolutionize a wide range of clinical applications, including risk stratification, diagnostics, monitoring, prevention, prognostics, and treatment selection. By moving beyond traditional statistical methods that seek latent space classifications, our mechanistic modeling approach builds on the underlying biological processes, offering deeper insights into disease mechanisms. This not only aids in identifying new therapeutic targets but also enables the safe comparison of multiple therapies through simulation. Moreover, digital twins can complement in vitro and in vivo experimentation by prioritizing targets and predicting efficacy before laboratory testing. Trust in model predictions is enhanced through transparency and explainability, as mechanistic models provide both confidence levels and detailed explanatory mechanisms. Fully addressing all these aspects is critical, and the community has already started to do so^10,148^. While acknowledging their importance as critical areas for future research and adoption of the methodology, our complementary focus here is to show that the complex systems methodology can help integrate multiscale, multiomics, and exposome data to design hypothesis-driven digital twins based on data-driven generative models.
